# Ictal semiology of epileptic seizures with insulo-opercular genesis

**DOI:** 10.1007/s00415-021-10911-0

**Published:** 2021-11-23

**Authors:** Eva Martinez-Lizana, Armin Brandt, Niels A. Foit, Horst Urbach, Andreas Schulze-Bonhage

**Affiliations:** 1 Epilepsy Center, Medical Center, University of Freiburg, Breisacher Str. 64, 79106 Freiburg im Breisgau, Germany; 2 Department of Neurosurgery, Medical Center, University of Freiburg, Freiburg im Breisgau, Germany; 3 Department of Neuroradiology, Medical Center, University of Freiburg, Freiburg im Breisgau, Germany

**Keywords:** Insulo-opercular epilepsy, Semiology, Epilepsy surgery, Video-EEG, Insular cortex

## Abstract

**Objective:**

Epileptic seizures with insular genesis are often difficult to distinguish from those originating in the temporal lobe due to their complex and variable semiology. Here, we analyzed differentiating characteristics in the clinical spectrum of insulo-opercular seizures.

**Methods:**

Ictal semiology in patients with a diagnosis of insulo-opercular epilepsy (IOE) based on imaging of epileptogenic lesions or electrophysiological evidence of an insulo-opercular seizure origin was retrospectively analyzed and compared to age-matched controls with mesial temporal lobe epilepsy (MTE).

**Results:**

Forty-six IOE and 46 matched MTE patients were included. The most prominent ictal features in IOE were focal motor phenomena in 80.4% of these patients. Somatosensory sensations, version, tonic and clonic features, when present, were more frequent contralateral to the SOZ in MTE patients, while they occurred about equally often ipsilateral and contralateral to the SOZ in IOE patients. Ipsilateral manual automatisms were significantly more frequent in MTE patients than in IOE (*p* = 0.010). Multivariate analysis correctly identified IOE in 78.3% and MTE in 84.8% using five semiologic features (Chi-square = 53.79 with 5 degrees of freedom, *p* < 0.0001). A subanalysis comparing patients with purely insular lesions with MTE patients using only the earliest ictal signs showed that somatosensory sensations are significantly more frequent in insular epilepsy (*p* = 0.010), while automatisms were significantly more frequent in MTE patients (*p* = 0.06).

**Significance:**

Our study represents the first in-depth analysis of ictal semiology in IOE compared to MTE. Use of these differentiating characteristics can serve for a correct syndrome classification and to steer appropriate diagnostic and local therapeutic procedures.

**Supplementary Information:**

The online version contains supplementary material available at 10.1007/s00415-021-10911-0.

## Introduction

Among focal epilepsies, insulo-opercular epilepsy (IOE) poses particular problems in its identification and treatment. The insular cortex is integral part of multiple functional systems, ranging from sensory processing to autonomic and motor control, with widespread connectivity [1, 2]. Epileptic activity may thus result in multifaceted clinical appearance of seizures, rendering a differentiation from seizures of temporal origin a problem [3, 4]. The electrophysiological access to insular discharges is severely hampered: covered by the opercula, scalp EEG does not provide direct recordings from the insular cortex, and only activity propagated to the dorsolateral convexity can be seen. Even seizures may lack a well localizable EEG correlate, which has led to confusion with non-epileptic, psychogenic seizures [5, 6]. Intracranial recordings need the use of depth electrodes; their implantation does carry an increased risk due to the multiple branches of the middle cerebral artery. Thus, most studies using SEEG are limited by severe undersampling of the insular cortex with an average of only 1–18 electrode contacts in the complete lobe in publications from Afif [7], Isnard [8, 9] and Blauwblomme [10]. In recent studies increased coverage of the insular cortex has been achieved, but the identification of potentially epileptogenic lesions on MR imaging often still constitutes the best evidence for seizure generation in the insular cortex; like in other localizations, however, even best available imaging techniques do not reveal a lesion in all patients [11, 12].

An improved understanding of semiological features of insulo-opercular epilepsy thus remains essential to raise suspicion for this syndrome. Unfortunately, the patient numbers included in previous reports on insulo-opercular seizure semiology have frequently been low [13–15]. According to previous reports, ictal semiology of insulo-opercular seizures comprises various non-specific patterns including somatosensory/viscerosensory, autonomic, speech-related abnormalities, fear and a variety of motor signs including automatisms [16, 17]. Importantly, these semiological features are not exclusive to IOE but are also found in frontal and temporal lobe epilepsy.

To date, no study has compared IOE to other common forms of focal epilepsy, although there is a clear need to improve identification of potential epilepsy surgery candidates or identify appropriate targets for focal stimulation. The differentiation of insulo-opercular seizures from TLE represents the most frequently encountered challenge, as an important fraction of patients who failed temporal lobe surgery may have been misdiagnosed with IOE or feature an extension of the epileptogenic zone into the insula [13, 18].

A better understanding of the neuroanatomical basis of insular ictal semiology will therefore likely improve candidate selection for resective procedures or electrode placement [3, 5, 19]. The purpose of this study is to assess seizure semiology of IOE more precisely and to identify factors/patterns segregating IOE from mesiotemporal epilepsy (MTE).

## Methods

Forty-six patients with a diagnosis of IOE at the Freiburg Epilepsy Center between 2003 and 2019 were retrospectively identified from medical records. The inclusion of patients in the IOE group was based on either MR evidence of an insular lesion which was regarded as typically epileptogenic (e.g., cortical dysplasia, tumors, cavernomas) and concordant information from scalp-EEG (fronto-temporal ictal patterns) or if there was stereo-electroencephalography (SEEG) evidence for insulo-opercular seizure generation (in two non-lesional cases and in additional ten patients with multiple lesions or lesions extending beyond the insular border to ascertain insulo-opercular seizure onset). A part of the epileptogenic lesions extended beyond the strict limits of the insular cortex in the adjacent opercula.

This IOE cohort was compared to 46 age-matched patients with previous successful selective amygdalohippocampectomy for drug-refractory mesial temporal lobe epilepsy (MTE) (Engel I outcome at a minimum of 12 months after surgery). Temporal lobe epilepsy was selected as control group as insulo-opercular seizure origin has been found to be a relevant cause of outcome failures of temporal resection [4].

Standardized clinical evaluation included detailed history and physical examination, MRI and long-term video-EEG monitoring (VEEG). Additionally, SEEG provided evidence of insular origin in 12 IOE patients (2 non-lesional cases; 1 patient with left hippocampal sclerosis and focal cortical dysplasia involving left insular and adjacent frontal and temporal cortex; 3 patients with an FCD involving insular and adjacent frontal and temporal regions; 1 patient with an extensive FCD involving insular and adjacent temporal and parietal regions; 1 patient with multiple dysplastic regions of left insula, right angular gyrus and right frontal cortex, 1 patient with tuberos sclerosis and tubers left fronto-insular, in cingulate cortex anterior, in frontal superior and medius gyrus; 1 patient with a cavernous hemangioma in the right putamen and a right temporo-insular ganglioglioma WHO grad I; 1 patient with a purely left insular FCD yet more extended ictal EEG patterns, findings on FDG-PET and ictal SPECT, and 1 patient with fronto-insular FCD who did not achieve seizure-freedom after a first surgery). Seven of the MTE patients with electroclinical discordance had undergone SEEG. MRI was performed with 1.5 T (Siemens Magnetom Vision) or 3 T scanners (Siemens Magnetom Trio or Prisma). T1-weighted sequences with and without gadolinium-diethylenetriaminepentaacetic acid (DTPA), T2w, fluid-attenuated inversion recovery (FLAIR) and magnetization-prepared rapid gradient echo (MPRAGE) sequences were obtained. To assess mesiotemporal lobe integrity, T2w axial and coronal images with a modified angulation parallel to the long axis of the hippocampus were acquired. Patients initially underwent VEEG at the Epilepsy Centers of Heidelberg, Kehl-Kork or Freiburg and were later referred to the University Medical Center Freiburg for presurgical evaluation and intracranial recordings, if necessary SEEG implantation was guided by non-invasive EEG data and covered the hypothetical epileptogenic zone(s) as well as the lesional area, if identified. All data were retrospectively assessed by review of medical records and video-EEG recordings.

Semiological features were analyzed at the patient level and classified in accordance with the latest operational classification of seizure types by the International League Against Epilepsy [20] into: somatosensory (including perioral and throat sensations), olfactory, auditory, gustatory, cephalic or epigastric sensation, déjà-vu, fear, autonomic alterations (including feeling of warmth, flush, ictal tachycardia or bradycardia, hypersalivation and nausea), aphasia, ictal speech, hyperkinetic or focal motor activity (including versive, clonic or tonic posturing), automatisms (oral or manual), behavioral arrest, and postictal aphasia. Focal seizures progressing to bilateral tonic–clonic seizures were also documented. Somatosensory sensations, manual automatisms, versive, clonic, and tonic activity were additionally classified into not present, present but non-lateralizing (bilateral or alternating between right and left), ipsi- or contralateral to the seizure onset zone (SOZ). Semiological features were considered initial if they were the earliest feature in at least one seizure of a given patient. In the case that different seizures had more than three different initial semiological signs, the three features most frequently occurring at the start of seizures were used for analysis.

A combined analysis was performed using lateralizing features expected to be contralateral to the SOZ (somatosensory sensations, versive, clonic, and tonic movements). The score was considered ipsilateral if at least one element was lateralized ipsilateral and none contralateral; contralateral, if at least one element was lateralized contralateral and none ipsilateral; and non-lateralizing, if at least one ipsilateral and one contralateral involvement was present, respectively.

Data analysis was performed using R version 4.0.2 [21]. Frequencies of semiological features were compared between IOE and MTE patients using Fisher's exact test (2-tailed). Features showing significantly different frequencies (*p* < 0.05) were included in an initial logistic regression model. As the data showed quasi-complete separation [22], a bias-reduced general linear model (R-package ‘brglm2’, version 0.6.2 [23]) was used for this purpose. Variables without a significant independent contribution to the initial multivariate model were then excluded by backward elimination.

To systematically identify co-occurring features, we performed a hierarchical agglomerative cluster analysis using complete linkage [24]. The distance between pairs of features was computed as the number of patients who exhibited different behavioral patterns for these features (one present and one absent) divided by the total number of patients (46 in each group).

**Fig. 1:**
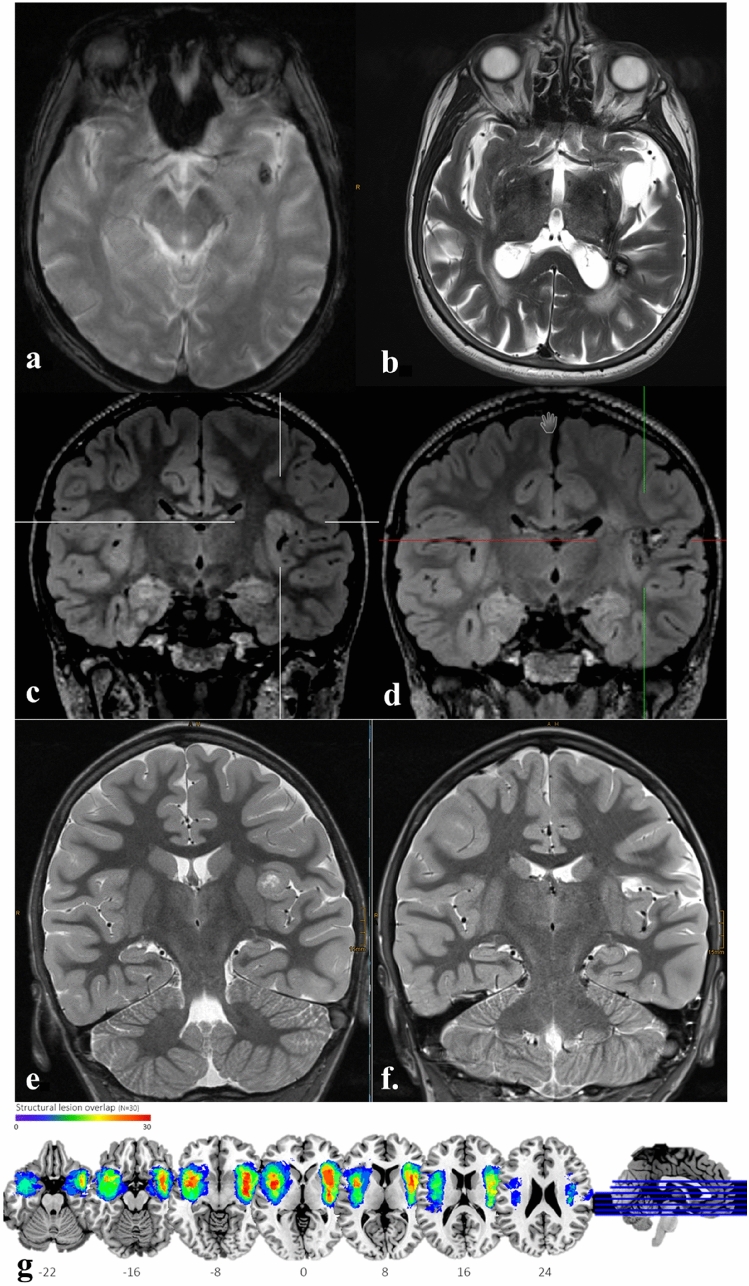
Examples of patients included based on a visible epileptogenic lesion: **a**, **b** Cavernoma in the left insula. Axial T2-MRI imagines of the lesion prior to epilepsy surgery (**a**) and following lesionectomy (**b**). **c**, **d** Focal cortical dysplasia in the left insula. Coronal T2-MRI imagines of the lesion prior to epilepsy surgery (**c**) and following lesionectomy (**d**); **e**, **f** Angiocentric glioma WHO I in the left anterior insula. Coronal T2-MRI imagines of the lesion prior to epilepsy surgery (**e**) and following lesionectomy (**f**). **g** From the 44 patients with a MR-based evidence for insular seizure origin, we performed heat map of lesion extension in patients with 3D MRI datasets available (*n* = 30). Individual binary lesion maps are superimposed onto the MNI152—template. Spectral colors demonstrate degrees of structural lesion overlap from violet = 1 patient to red = all patients in the cohort. Note that in some patients, the lesion extended into the adjacent operculum

To further investigate lesion-semiology associations, an expert rater (NAF) manually segmented structural lesions on individual volumetric T1w MRI in native space with ITK-snap for patients in which volumetric imaging data were available (*n* = 30) [25]. Following structural pre-processing of individual T1w images including segmentation using SPM12 (fil.ion.ucl.ac.uk/spm) default routines, binary lesion maps were normalized to Montreal Neurological Institute (MNI) stereotaxic space with the transformation matrices obtained during preprocessing. Group-specific lesion maps were created with MRIcroGL [26] (Fig. 1a).

The study was approved by the Ethics Review Board of the University Medical Center Freiburg and the patients gave their written consent that the data collected for presurgical epilepsy diagnosis may be used for scientific evaluation.

## Results

This series consists of 46 patients with IOE (27 women, mean age at evaluation 28 years; mean age at onset 16 years, mean duration of epilepsy 12 years) and 46 MTE patients with a lesion in the same hemisphere and approximately (mean 31 years) the same age at presurgical workup. Further demographic and clinical data are summarized in Table 1. Based on the electroclinical and imaging data, seizures originated in the right hemisphere in 26 patients and in the left hemisphere in 20 patients.

**Table 1:** Demographics and clinical findings in patients with insulo-opercular epilepsy and the matched-pair control patients with mesiotemporal epilepsy

Characteristics	Insulo-opercular epilepsy patients (*n* = 46)	Mesiotemporal epilepsy patients (*n* = 46)
Sex (male/female)	19/27	19/27
Age at epilepsy onset (years) mean/median	16.1/12.5	13.5/10.0
Duration of epilepsy, years		
Mean	12	18
Range	0–40	0–43.4
Lesion on MRI, *n* (%)	44 (95.7)	46 (100)
Cavernoma	2	1
DNET	2	0
FCD	21	3
Gliosis	1	0
Infarction	1	0
Hippocampal Sclerosis	0	36
Tuber	2	0
Other tumors	17	6

In IOE patients, focal motor phenomena were the most frequent ictal semiological manifestation (in 80.4% of patients), including tonic posturing in 63.0%, clonic movements in 58.7%, version in 23.9% of patients, and hyperkinetic features in 21.7%. Behavioral arrest occurred in 54.3% of the patients, oral automatisms in 43.5% and manual automatisms in 39.1%. Autonomic signs and symptoms were reported in 58.7% of the patients, including feeling of warmth in 13.0%, flush in 19.6%, ictal tachycardia/bradycardia in 30.4% and nausea in 8.7%. Somatosensory focal aware seizures were recorded in 32.6%, olfactory or gustatory sensations in 17.4%, auditory sensations in 8.7%, déjà-vu in 8.7%, fear in 10.9%, cephalic in 21.7% and epigastric sensations in 19.6% of the patients. Ictal or postictal aphasia was present in 32.6% and ictal speech in 4.3% of the patients.

When comparing with MTE patients, somatosensory symptoms were significantly more frequent in patients with IOE (32.6% vs. 10.9%, *p* = 0.021), as well as focal motor phenomena (80.4% vs. 52.2%, *p* = 0.008) and hyperkinetic features (21.7% vs. 0%, *p* = 0.001). Auditory sensations were found only in IOE, not in MTE, whereas olfactory or gustatory sensations were found in both IOE (17.4%) and MTE (6.5%). In contrast, epigastric sensations were more frequent in MTE patients (19.6% vs. 50.0%, *p* = 0.004), as were ictal automatisms (54.3% vs. 89.1%, *p* < 0.001), behavioral arrest (54.3% vs. 84.8%, *p* = 0.003) and ictal speech (4.3% vs. 26.1%, *p* = 0.007). Autonomic features were observed in a high percentage of both groups. Particularly, feeling of warmth was found in 2 patients with MTE and 6 patients with IOE, flush in 6 patients with MTE and 9 patients with IOE, ictal tachycardia or bradycardia in 17 patients with MTE and 14 patients with IOE, hypersalivation in 1 patient with MTE and 5 patients in IOE and nausea in 6 patients with MTE and 4 patients with IOE. Evolution to bilateral tonic–clonic seizures tended to appear more frequently in patients with MTE (67.4%) than in patients with IOE (52.2%). Results of univariate analyses of the semiologic features of IOE and MTE patients are shown in Table 2.

**Table 2:** Semiologic features during seizures of patients with insulo-opercular epilepsy or mesiotemporal epilepsy

Semiologic feature	Insulo-opercular epilepsy, *n* (%) *n* = 46	Mesiotemporal epilepsy, *n* (%) *n* = 46	*p* value (two-tailed Fisher’s exact test)
Somatosensory	15 (32.6)	5 (10.9)	0.021
Olfactory or gustatory	8 (17.4)	3 (6.5)	0.117
Auditory	4 (8.7)	0 (0)	0.197
Déjà-vu	4 (8.7)	0 (0)	0.117
Fear	5 (10.9)	4 (8.7)	1.000
Cephalic	10 (21.7)	7 (15.2)	0.592
Epigastric	9 (19.6)	23 (50.0)	0.004
Autonomic	27 (58.7)	35 (76.1)	0.119
Ictal or postictal aphasia	15 (32.6)	25 (54.3)	0.058
Ictal speech	2 (4.3)	12 (26.1)	0.007
Automatisms	25 (54.3)	41 (89.1)	< 0.001
Hyperkinetic	10 (21.7)	0 (0)	0.001
Focal motor	37 (80.4)	24 (52.2)	0.008
Behavioral arrest	25 (54.3)	39 (84.8)	0.003
Evolution to BTCS*	24 (52.2)	31 (67.4)	0.202

*BTCS bilateral tonic–clonic seizure

Patients with purely insular lesions (*n* = 15) were separately analyzed and compared with the MTE patients (Supplementary Table 1). Similarly to the previous analysis, somatosensory symptoms were significantly more frequent in patients with insular epilepsy (IE) (47.6% vs. 10.9%, *p* = 0.006). Focal motor phenomena (80% vs. 52.2%) remained more frequent in patients with IE than in MTE, but the difference was no longer significant. Still in the subgroup with 15 patient with IE there were auditory sensations and hyperkinetic features but not in MTE, whereas olfactory or gustatory sensations occurred in both IOE (20%) and MTE (6.5%). Ictal automatisms were more frequent in MTE patients than in IE patients (89.1% vs. 40%, as were instances of behavioral arrest (84.8 vs. 33.3% *p* < 0.001). Ictal speech was present only in MTE (26.1%). Epigastric sensations tended to be more frequent in MTE patients than in IE patients (50% vs. 26.7%) but this difference was not significant.

When taking into account only the earliest (up to three) ictal signs of patients with purely insular lesions and patients with MTE, respectively, somatosensory symptoms were significantly more frequent in patients with IOE than in patients with MTE. In contrast, Automatisms were more frequent in MTE patients. Initial focal motor phenomena appeared in 47% of patients with purely insular lesions and in 22% of patients with MTE (Supplementary Table 2).

Results of the univariate analysis of lateralizing SF in IOE and MTE patients are shown in Table 3. Ipsilateral manual automatisms were significantly more frequent in MTE patients than in IOE (*p* = 0.010). We performed a combined univariate analysis with somatosensory sensations, version, tonic and clonic features (see “Methods”). If one or more of the features were present, they were predominantly present contralateral to the SOZ in MTE patients whereas in IOE patients, they occurred about equally often ipsilateral as contralateral to the SOZ (*p* = 0.0217, Table 3).

**Table 3:** Lateralizable semiologic features during seizures of patients with insulo-opercular epilepsy or mesiotemporal epilepsy

Lateralizing semiologic feature		Insulo-opercular epilepsy, *n* (%) *n* = 46	Mesiotemporal epilepsy, *n* (%) *n* = 46
Somatosensory	None	32 (67.4)	42 (89.1)
	Non-lateralizable	10 (21.7)	4 (8.7)
	Ipsilateral	3 (6.5)	1 (2.2)
	Contralateral	2 (4.3)	0 (0)
Manual automatisms	None	28 (60.9)	13 (28.3)
	Non-lateralizable	5 (10.9)	3 (6.5)
	Ipsilateral	7(15.2)	19 (41.3)
	Contralateral	6 (13.0)	11 (23.9)
Version	None	35 (76.1)	32 (69.6)
	Non-lateralizable	3 (6.5)	2 (4.3)
	Ipsilateral	3 (6.5)	1 (2.2)
	Contralateral	5 (10.9)	11 (23.9)
Clonic	None	26 (56.5)	29(63.0)
	Non-lateralizable	7 (15.2)	9 (19.6)
	Ipsilateral	6 (13.0)	2 (4.3)
	Contralateral	7 (15.2)	6 (13.0)
Tonic	None	17 (37.0)	22 (47.8)
	Non-lateralizable	11 (23.9)	8 (17.4)
	Ipsilateral	9 (19.6)	1 (2.2)
	Contralateral	9 (19.6)	15 (32.6)
Combined analysis with Somatosensory, version, tonic and clonic	None	6 (13.0)	16 (34.8)
	Non-lateralizable	19 (41.3)	8 (17.4)
	Ipsilateral	10 (21.7)	3 (6.5)
	Contralateral	11 (23.9)	19 (41.3)

Ipsilateral manual automatisms are significantly more frequent in patients with mesiotemporal epilepsy than in patients with insulo-opercular epilepsy (*p* = 0.010). The combined analysis with somatosensory, version, tonic and clonic symptoms showed that if one or more of the features were present, were predominantly present contralateral to the lesion in mesiotemporal patients whereas in insular patients, they occurred about equally often ipsilateral as contralateral to the lesion

Results of a logistic regression analysis including semiologic features with a *p*-value < 0.05 in the univariate analysis is shown in Table 4. The *χ*^2^ for the overall likelihood ratio test of the model was 64.1 (*p* < 0.0001). Variables with an odds ratio greater than 1 are independently predictive for IOE (hyperkinetic features). On the other hand, variables with an odds ratio less than 1 are independently predictive for MTE (epigastric sensations, aphasia, ictal speech and automotor features). Table 4 shows the reclassification accuracy when using the final multivariate logistic regression model. Using this model containing five features with significant contributions (epigastric sensations, aphasia, ictal speech, automotor and hyperkinetic or motor features), 78.3% of IOE patients and 84.8% of MTE patients were correctly identified.

**Table 4:** Multivariate logistic regression analysis of semiologic features that best differentiated between patients with insulo-opercular epilepsy and patients with mesiotemporal epilepsy

Semiologic feature	Odds ratio	95% Confidence interval of odds ratio	*p* value
Epigastric	0.303	0.094–0.980	0.046
Aphasia	0.313	0.104–0.941	0.039
Ictal speech*	0.021	0.001–0.433	0.012
Automotor	0.144	0.037–0.562	0.005
Hyperkinetic*	275.941	3.231–23,567.469	0.013

Patients	Correct		Incorrect	
	No	%	No	%
Insulo-opercular epilepsy	36	78.3	10	21.7
Mesiotemporal epilepsy	39	84.8	7	15.2

Variables with an odds ratio greater than 1 are independently predictive for an IE. Variables with an odds ratio less than 1 are independently predictive for a MTE. Using this model with the five significant features 78.3% of insular patients and 84.8% of mesiotemporal patients were correctly identified

*Estimation of the odds ratio for the features ‘Hyperkinetic’ and ‘Ictal speech’ is unreliable, due to quasi-complete separation of the data set [41]

**Fig. 2:**
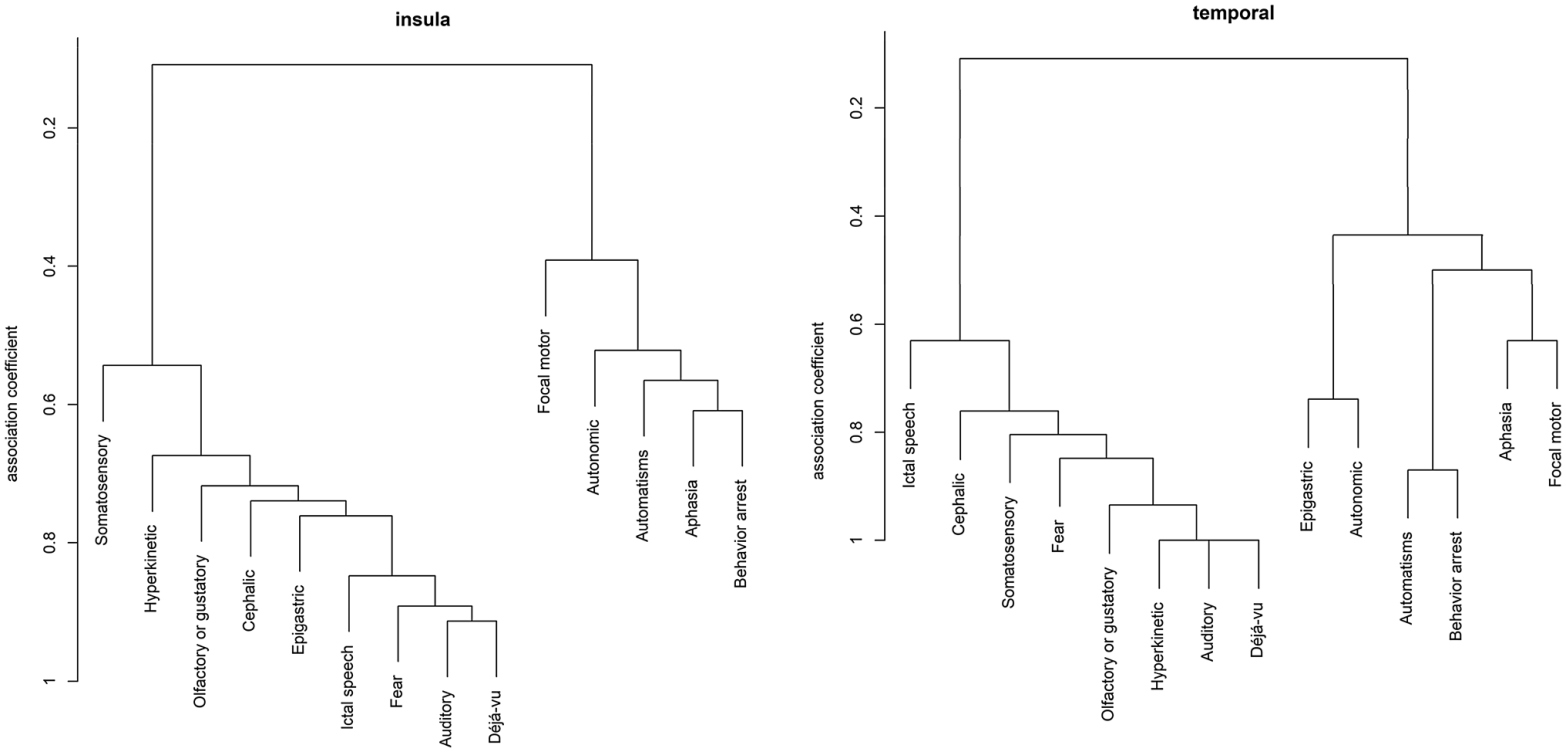
Results of the cluster analysis. The left dendrogram shows features of the patients with insulo-opercular epilepsy and on the right features of the patients with mesiotemporal epilepsy

Cluster analysis results are shown in Fig. 2 as dendrograms. While we could identify two separate clusters of symptoms in both IOE and MTE patients, feature associations differed between the two groups. In patients with IOE, cluster 1 was characterized by déjà-vu, auditory sensations, fear, ictal speech, cephalic and epigastric sensations, olfactory or gustatory sensations, somatosensory symptoms and hyperkinetic features. Cluster 2 was comprised of behavioral arrest, aphasia, automatisms, autonomic and focal motor features. In patients with MTE, the first cluster was characterized by déjà-vu, hyperkinetic features, olfactory, gustatory or auditory sensations, fear, somatosensory symptoms, cephalic sensations and ictal speech, while the second cluster was characterized by three sub-clusters consisting of behavioral arrest and automatisms, autonomic symptoms and epigastric sensations, and focal motor features and aphasia, respectively.

## Discussion

We here analyzed the semiology of a large cohort of patients with insulo-opercular seizure generation. Evidence for the insula was based on an insular lesion in 34/46 cases, on an insular lesion and additional SEEG confirmation in 10/46 cases, and on SEEG in two non-lesional cases. As mentioned above, SEEG is limited in providing evidence of an insular origin due to an undersampling of the insular cortex with standard lateral approaches; it was thus implemented only in multilesional cases (e.g., tuberous sclerosis), when the limits of the lesion were not clear (e.g., some FCD) or in non-lesional cases.

Interestingly, the results of our study found seizure characteristics, which highly significantly allowed to separate the insulo-opercular seizures from mesiotemporal seizures. This supports the localizing role of semiologic features in presurgical evaluation [27–31]. In particular, the data may be helpful in pointing towards seizure generation beyond the temporal lobe.

Corroborating previous work, our study clearly demonstrates that insulo-opercular seizure semiology is very heterogeneous [31, 32]. Presence of focal motor phenomena was the most frequent semiological feature in agreement with one previous study [33], suggesting early spread from the insula to the primary motor cortex, premotor cortex and the frontal pole. This finding is in concordance with a systematic review on published cases of insular seizures [34]. The second most frequent seizure manifestation was somatosensory (including perioral and throat sensations) and autonomic symptoms (including bradycardia) [16, 17, 34]. Progression to bilateral tonic–clonic seizures was less frequent in other series than in our cohort, which may be partially related to tapering of antiepileptic drugs during video-EEG recordings in our patient sample.

Somatosensory symptoms were present significantly more frequently in IOE than MTE. Focal motor phenomena were only found significantly more frequently in IOE than MTE when analyzing all semiological signs. In the analysis of the earliest ictal signs, the difference was no longer significant. Our data support the hypothesis that in insular epilepsy, focal motor features more likely reflect activation of fronto-insular networks rather than being primarily insular seizure manifestations, in agreement with the studies of Singh and Isnard [2, 3].

In addition, we found auditory sensations in 8.7% of IOE; this is of interest and reflects the involvement of insula in auditory processing [35]. Of note, auditory symptoms were not present in the temporal cohort reflecting the strictly mesiotemporal seizure origin; this differentiation cannot be extended to temporal neocortical epilepsy which may involve the auditory cortex and belt. An interesting point is that IOE seizures had a lower lateralizing features compared to temporal lobe seizures. The lower lateralizing value of the ictal features in patients with IOE may be explained through the wide connectivity of the insula, including contralateral brain regions [36], possibly reflecting early contralateral propagation of the ictal activity [32] and bilateral representation within the insular cortex [37].

Hyperkinetic phenomena was the strongest predictive features for patients with IOE in the multivariate analysis. We observed hyperkinetic features in IOE in 21.7% of patients, which is similar as reported by Peltola et al. and Wang et al. [16, 17]. This was the most distinguishing feature from a mesiotemporal onset. Of note, also temporal neocortical seizures arising from the temporal pole may present with hyperkinetic motor phenomena [38]. While we also found autonomic symptoms in a high proportion of patients with IOE (58.7%) (see also [16]); their presence, however, did not separate IOE from MTE in which autonomic symptoms and signs were found in 76.1%. Moreover, epigastric sensations and automatisms were significantly more frequent in MTE compared to IOE; and epigastric sensations, automatisms, ictal speech and aphasia were independently predictive of MTE in the multivariate analysis. In a model encompassing the features hyperkinetic, epigastric, aphasia, ictal speech and automatism, 78.3% of IOE patients and 84.8% of MTE patients were identified correctly. Previous studies with intracranial electrodes showed that there are distinct semiological subgroups in the insula [16, 31, 32]. This study did not separate insular subregions but joined semiological features from all IOE cases for differentiation from mesiotemporal onset. This reflects a variable extension of lesions within the insular cortex and connectivity within the insula and provides clinically relevant information for presurgical localization and planning of surgery.

The performed cluster analysis revealed that in both patient groups, subjective feelings (including somatosensory sensations but also fear, olfactory, gustatory, auditory, déjà-vu or cephalic sensations) and hyperkinetic features were associated in one cluster, behavioral arrest, aphasia, automatisms, autonomic features, and focal motor features in a second one. Ictal speech characterizes seizures generated in the non-dominant hemisphere, which may contribute to its positive association with reported subjective feelings in both patient groups, as awareness is more frequently preserved and subjective feelings thus experienced during and remembered after the seizure. We did not find obvious relationship between the semiological subclusters and anatomic subregions of the insula. This may reflect epileptogenic lesions extending not limited to a subarea of the insula but rather across the whole insula as well as a role of ictal propagation in the clinical manifestation of the seizures.

The insula is by no means an isolated functional center, as the term “insula” may suggest, rather the wide clinical spectrum of insular seizures reflects that the insula acts as a multimodal hub region with extensive structural and functional connections to other brain regions [36]. On the other hand, the anatomical organization of the insula per se is highly heterogeneous and its disruption by seizures may similarly elicit a wide spectrum of symptoms [2, 7]. While epigastric, olfactory, gustatory and auditory sensations may relate to a primary generation within the insular cortex [39, 40], behavioral arrest and automatisms likely reflect seizure spread through extensive connections to the temporal lobe; emotional and cognitive auras to the limbic regions; tonic, clonic or hyperkinetic phenomena and speech dysfunction to frontal regions [1].

The semiological features reported here, as well as the stereotypy of events, imaging and the knowledge that at least focal aware seizures may not be accompanied by scalp EEG correlates will also contribute to a better clinical judgement and differentiation between PNES and seizures of insular origin.

This study has several limitations. Some patients had a lesion which extended beyond the strict borders of the insula, particularly to one of the opercula. This reflects the clinical spectrum of patients presenting with insular seizures and was thus not chosen as an exclusion criterion. An extension of the SOZ beyond the peri-insular circular sulcus may favor the occurrence of motor seizures. Future prospective multicenter assessments in larger groups of patients may provide further insight of the role of adjacent regions for the newly identified semiological clusters, including the temporal sequence of symptoms, and the distinction of IOE to other regions of focal epileptogenesis.

## Supplementary Information

Below is the link to the electronic supplementary material.

Supplementary file1 (DOCX 26 KB)
