# Supplementary material for: Ictal semiology of epileptic seizures with insulo-opercular genesis
Source: J Neurol. 2021 Nov 23;269(6):3119–28. doi: 10.1007/s00415-021-10911-0 (PMC9120119; doi:10.1007/s00415-021-10911-0)
Supplement: Supplementary file 1 — Supplementary file1 (DOCX 26 KB) [file 415_2021_10911_MOESM1_ESM.docx]

**Supplementary Information:**

| **Semiologic feature** | **Purely Insular epilepsy, n (%) n=15** | **Mesiotemporal epilepsy, n (%)  n=46** | **p  (2-tailed Fisher’s exact test)** |
| --- | --- | --- | --- |
| Somatosensory | 7 (46.7) | 5 (10.9) | **0.006** |
| Olfactory or gustatory | 3 (20.0) | 3 (6.5) | 0.152 |
| Auditory | 2 (13.3) | 0 (0) | 0.057 |
| Déjà-vu | 1 (6.7) | 0 (0) | 0.246 |
| Fear | 1 (6.7) | 4 (8.7) | 1.000 |
| Cephalic | 3 (20.0) | 7 (15.2) | 0.696 |
| Epigastric | 4 (26.7) | 23 (50.0) | 0.143 |
| Autonomic | 9 (60.0) | 35 (76.1) | 0.320 |
| Ictal or postictal aphasia | 4 (26.7) | 25 (54.3) | 0.079 |
| Ictal speech | 0 (0.0) | 12 (26.1) | **0.028** |
| Automatisms | 6 (40.0) | 41 (89.1) | **<0.001** |
| Hyperkinetic | 1 (6.7) | 0 (0) | 0.246 |
| Focal motor | 12 (80.0) | 24 (52.2) | 0.074 |
| Behavioral arrest | 5 (33.3) | 39 (84.8) | **<0.001** |
| Evolution to BTCS* | 6 (40.0) | 31 (67.4) | 0.074 |
| **BTCS=bilateral tonic-clonic seizure*  **Supplementary table 1.** Semiologic features during seizures of patients with insular epilepsy or mesiotemporal epilepsy. | | | |

| **Initial Semiologic feature** | **Purely Insular epilepsy, n (%) n=15** | **Mesiotemporal epilepsy, n (%)**  **n=46** | **p**  **(2-tailed Fisher’s exact test)** |
| --- | --- | --- | --- |
| Somatosensory | 6 (40.0) | 4 (8.7) | **0.010** |
| Olfactory or gustatory | 1 (6.7) | 1 (2.2) | 0.434 |
| Auditory | 1 (6.7) | 0 (0.0) | 0.246 |
| Déjà-vu | 1 (6.7) | 0 (0.0) | 0.246 |
| Fear | 0 (0.0) | 1 (2.2) | 1.000 |
| Cephalic | 1 (6.7) | 2 (4.3) | 1.000 |
| Epigastric | 1 (6.7) | 15 (32.6) | 0.087 |
| Autonomic | 2 (13.3) | 7 (15.2) | 1.000 |
| Ictal or postictal aphasia | 1 (6.7) | 6 (13.0) | 0.670 |
| Focal motor | 7 (46.7) | 10 (21.7) | 0.096 |
| Automatisms | 2 (13.3) | 26 (56.5) | **0.006** |
| Behavioral arrest | 5 (33.3) | 6 (13.0) | 0.118 |
| **Supplementary table 2.** Analysis of the earliest ictal signs (up to three) in patients with insular epilepsy and in patients with mesiotemporal epilepsy. | | | |
